# Insights of Photocatalytic Properties of Fe/TiO_2_ Bio-Based Particles: Experimental and Modeling Design Toward Methyl Orange Photodegradation

**DOI:** 10.3390/e28060632

**Published:** 2026-06-03

**Authors:** Aleksandar Jovanović, Amil Aligayev, Mladen Bugarčić, Dimitrije Anđić, Ulkar Samadova, Jelena Dimitrijević, Miroslav Sokić, Qing Huang

**Affiliations:** 1 Institute for Technology of Nuclear and Other Mineral Raw Materials, Boulevard Franše d’Eperea 86, 11000 Belgrade, Serbia; m.bugarcic@itnms.ac.rs (M.B.); d.andjic@itnms.ac.rs (D.A.); j.dimitrijevic@itnms.ac.rs (J.D.); m.sokic@itnms.ac.rs (M.S.); 2 NOMATEN Centre of Excellence, National Centre for Nuclear Research, Andrzeja Sołtana 7/3, 05-400 Otwock, Poland; amil.aligayev@ncbj.gov.pl; 3 Scientific Research Center, Baku Engineering University, H. Aliyev, Khirdalan, AZ0101 Baku, Azerbaijan; 4 Milan Blagojević-Namenska AD, mr Radoša Milovanovića 2A, 32240 Lučani, Serbia; 5 Institute of Physics of the Ministry of Science and Education of the Republic of Azerbaijan, 131 H. Javid Avenue, AZ1073 Baku, Azerbaijan; ulkar.samadova@adda.edu.az; 6 Institute of Health and Medical Technology, Hefei Institutes of Physical Science, Chinese Academy of Sciences, Hefei 230031, China; huangq@ipp.ac.cn

**Keywords:** sustainable photocatalysis, green photocatalysts, kinetics, dye decomposition, DFT

## Abstract

This study investigates the electronic and photocatalytic properties of greenly fabricated rutile-phase titanium dioxide (bTiO_2_) modified with iron vanadate (Fe/bTiO_2_/VO_4_) and vanadium-substituted goethite (Fe/bTiO_2_/VOOH) by detailed experimental assay and density functional theory (DFT) calculations. Our analysis of the density of states (DOS), band structure, and work function reveals that both dopant systems significantly modify the electronic structure of pure rutile bTiO_2_. The dye methyl orange (MO) was used as the model pollutant. During photodegradation tests, parameters such as the reaction time, solid-to-liquid ratio, initial concentrations of the photocatalyst and dye, as well as distance of the lamp from the reactor and pH were varied. Degradation kinetics follows the equation of the pseudo-first order law for both photocatalysts (*k*_VO4_ = 0.058 min^−1^ and *k*_VOOH_ = 0.065 min^−1^), while degradation efficiencies of 92% and 99% were observed after 120 min at pH 3, respectively. Specifically, the DOS analysis highlights the contribution of Fe 3d and V 3d orbitals, which create new electronic states within the bandgap, facilitating charge transfer. These insights provide a strong foundation for the rational design of novel, highly efficient Fe/bTiO_2_-based photocatalysts for the degradation of organic pollutants in water.

## 1. Introduction

The textile sector solely releases more than 0.2 Mt/year of various dyes into the ecosystem, making water contamination a serious worldwide environmental concern [1]. Increased chemical (COD) and biological oxygen demand (BOD) values together with diminished illumination for aquatic plants might result from dye discharges. Many classes of dyes tend to persist in the ecosystem and build up in the food chain, which can have detrimental repercussions on organisms’ health [2,3]. Mostly, they are not biodegradable owing to intricate aromatic molecules, which are made to withstand different external conditions. Chemical dyes, especially azo types such as methyl orange (MO), are known for having harmful effects, mutational potential, and carcinogenicity potential [4,5,6]. MO is a common anionic azo dye that is mostly utilized as a coloring agent in the various branches of industries. Therefore, the concentrations of MO detected in some water streams are increasing [7,8].

Conventional processes like ionic exchange [9], adsorption [10], coagulation [11], filtration [12], and activated sludge biodegradation [13] have shown some prominent results toward dye elimination. The observed drawbacks of traditional methods [14] push forward scientists to propose novel treatment techniques. Hence, advanced oxidation processes (AOPs) like photocatalysis [15], a versatile technique, offer complete mineralization of the starter pollutant without transferring contaminants from one aggregate phase to another. Photocatalysis includes the activation of the photocatalyst’s surface under different types of radiation, with the generation of radical species helping in pollutant oxidation [16].

Different materials were employed as the photocatalyst, while TiO_2_-based materials show great performance. Given its high efficiency, durability, and affordability, TiO_2_ is regarded as one of the most significant photocatalysts [17]. Iron-modified TiO_2_ materials (Fe/TiO_2_) can be further advanced in order to exceed the confines of TiO_2_, particularly its inactivity beneath the visible light radiation. Fe/TiO_2_ photoactive materials are particularly powerful at breaking down different groups of organic contaminants, owing to their capacity to produce reactive oxygen species (•OH and •O^2−^). The insertion of Fe^3+^ into TiO_2_ matrices (due to the smaller ionic radius) could better explain the excitation process, where added ions permit photons from the visible range of sunlight to be caught by reducing the energy bandgap [18]. Given its relatively small bandgap (about 2.0–2.2 eV), enabling it to capture a wide range of the visible light spectrum, iron vanadate (FeVO_4_) is a semiconductor that has drawn a lot of notice [19]. Additionally, iron oxyhydroxide orthovanadate (FeVOOH) enhances the outstanding resilience of TiO_2_ by acting as a visible light-responsive receptor. A smart technological solution to get beyond the broad bandgap restrictions is the chemical alteration of TiO_2_ with FeVO_4_ or FeVOOH, creating a hybrid composite that efficiently degrades different pollutants. The positional distinction between photogenerated charge carriers is driven by this change, which produces a type-II heterojunction at the interface and a distorted band configuration [20]. In particular, the degree of recombination is greatly decreased as electrons move to the conduction band while holes build up in the valence band [21]. In addition to extending the edge of absorption towards the area of visible light, this collaborative effect increases the production of superoxide (•O^2−^) and hydroxyl (•OH) radicals, which results in greater efficacy in the oxidation of intricate chemical dyes [22].

A chain of redox phenomena triggered by light cause MO to degrade on a photocatalyst surface such as Fe/TiO_2_ [23].

The photocatalyst produces electrons (e^−^) and holes (h^+^) whenever it receives illumination from a light source:

(1) $$Fe/TiO_{2}+h\nu \to e^{-}+h^{+}$$

These produce potent radicals when they interact with oxygen and water:

(2) $$h^{+}+H_{2}O\to •OH+H^{+}$$

(3) $$e^{-}+O_{2}\to •O_{2}^{-}$$

The last part of the mechanism consists of two steps. A chain of redox processes initiated by light irradiation leads to the degradation of MO on the photocatalyst surface. The photogenerated charge carriers (e^−^ and h^+^) may participate in several parallel oxidative pathways. In particular, photogenerated holes (h^+^) may directly oxidize adsorbed dye molecules or react with surface hydroxyl groups and water to form hydroxyl radicals (•OH), while conduction band electrons may reduce dissolved oxygen to superoxide radicals (•O_2_^−^). The degradation of MO is therefore expected to proceed through a combination of direct hole oxidation and indirect radical-mediated pathways involving reactive oxygen species. These processes can lead to the cleavage of azo bonds (–N=N–) and subsequent transformation into smaller intermediate species. After that, the intermediate byproducts decompose, producing innocuous inorganic compounds:

(4) $$MO+•OH\to Intermediates\to CO_{2}+H_{2}O+SO_{4}^{2-}+NH_{4}^{+}+NO_{3}^{-}$$

**Figure 1:**
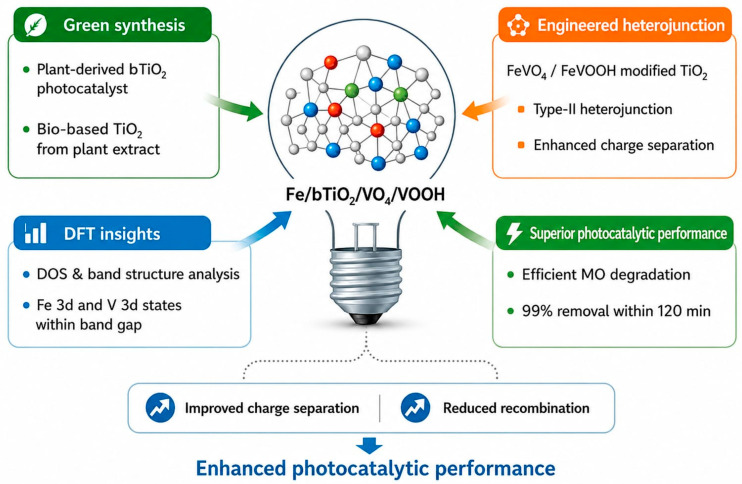
Graphically presented novelties of our study.

The goal of this study was to propose an efficient photodegradation system for the degradation of MO from wastewater samples. Therefore, two distinctive photoactive catalysts were fabricated (Fe/bTiO_2_/VO_4_ and Fe/bTiO_2_/VOOH) and morphologically investigated. The current synthesis system included a greater share of bio-based components such as bTiO_2_ from plant extract. Fabricated heterojunctions show promising results toward MO degradation. Furthermore, the hybrid structure provides better chemical stability and photoactivity compared to individual components, as the bTiO_2_ core acts as a robust scaffold that prevents the leaching of iron species. The main novelties of this study are presented in Figure 1.

## 2. Materials and Methods

### 2.1. Synthesis of Fe-Based Photocatalysts

In this photocatalytic study, two different photocatalysts were synthesized, following the modified procedure from Jovanovic et al. [24]. Instead of using commercial TiO_2_ as stated in previous work, for obtaining Fe-based photocatalysts, we used bio-based TiO_2_ fabricated following the procedure from our previous study [25]. Namely, sustainably developed TiO_2_ particles (bTiO_2_) from citrus peel extract and titanium isopropoxide (purity ≥ 99.5%, Thermo Fisher, Waltham, MA, USA) significantly contributes to the green production of particles due to the absence of toxic solvents and chemicals and lower temperatures. Titanium isopropoxide undergoes hydrolysis and subsequent condensation reactions to form Ti–O–Ti networks, leading to the formation of TiO_2_. The citrus peel extract, containing polyphenols and organic acids, may interact with titanium species through complexation and hydrogen bonding, acting as a natural stabilizing and structure-directing agent during particle formation. In conventional synthesis, chemical reducing and stabilizing agents (e.g., alkoxides, surfactants, or ammonia) are typically employed, whereas the citrus extract provides a greener alternative with similar functional roles [26,27]. The use of citrus peel extract enables a greener synthesis approach by replacing conventional chemical reagents such as synthetic reducing and stabilizing agents. Due to the presence of bioactive compounds (e.g., polyphenols, flavonoids, and organic acids), the extract can act as both a reducing and capping agent, facilitating the formation of the photocatalyst and influencing particle growth and stability [28,29].

Synthesis of both Fe-photocatalysts was performed in three-necked borosilicate reactors (500 mL), fitted with a refluxed condenser, air inlet nozzle, and thermocouple. The reactor was placed on a magnetic stirrer (IKA C-MAG HS7, Staufen, Germany), under the 200 rpm.

**Figure 2:**
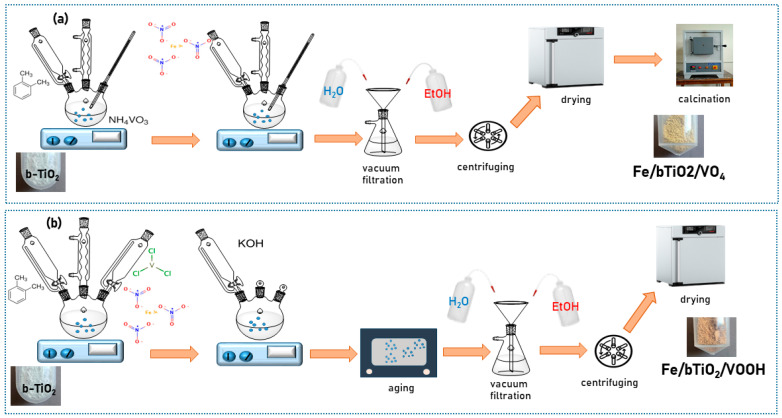
Route for the bTiO_2_-based photoactive composites’ fabrication: (**a**) Fe/bTiO_2_/VO_4_; (**b**) Fe/bTiO_2_/VOOH.

Synthesis of the first photocatalyst (FeVO_4_-bTiO_2_) consisted of consecutive steps (Figure 2a). First, 40 mL of deionized water (DW, 18.2 MΩ cm) was merged with 20 g of bTiO_2_ and 0.75 g of ammonium metavanadate (≥99.0 wt.%, Merck KGaA, Darmstadt, Germany). After covering the slurry via the addition of 120 mL xylene (≥75.0 wt.%, Merck KGaA, Darmstadt, Germany), the suspension was heated to 70 °C for one hour. The solution was then heated at 70 °C for one hour without mixing after 20 mL of Fe(NO_3_)_3_ × 9H_2_O (≥98 wt. %, Merck KGaA, Darmstadt, Germany) solution insertion, in order to allow for the initial interaction and surface deposition onto the bTiO_2_ support. Subsequently, the system was stirred to ensure complete homogenization of the mixture. The resulting material was then centrifuged, washed, and dried for six hours. The prepared particles (Fe/bTiO_2_/VO_4_) were then annealed for four hours at 500 °C.

Multiple chemical pathways were also involved in the production of the second photocatalyst, Fe/bTiO_2_/VOOH (Figure 2b). In a reactor, 20 g of bTiO_2_ particles and 100 mL of xylene were placed. Prior to heating at 70 °C for 1 h, in the reaction system, we induced a water solution formed of 7.5 g of VCl_3_ (97 wt.%, Merck KGaA, Darmstadt, Germany) and 4.3 g of Fe(NO_3_)_3_ × 9H_2_O. Following that, 15 mL of a 15 M KOH (≥85.0 wt.%, Merck KGaA, Darmstadt, Germany) solution was poured dropwise, and the mixture was agitated for another hour. In order to produce particles with reduced diameters, the produced solution was kept in the dark for 15 days. Finally, the solution was dried after being washed with ethanol and deionized water until there was a negative chloride reaction. The presence of chloride ions after synthesis was checked using a standard AgNO_3_ (99.0 wt.%, Merck KGaA, Darmstadt, Germany) test, where the formation of a white precipitate (AgCl) indicates the presence of chloride species [30].

### 2.2. Structural Characterization

The produced photocatalyst was structurally characterized by applying scanning electron microscopy with the energy dispersive spectroscopy (SEM-EDS) technique.

SEM (JEOL JSM-7001F, Tokyo, Japan) was used to assess the surface morphology following bTiO_2_ alteration with Fe-dopants. To ascertain the distribution of elements within the photocatalyst, the EDS (Oxford Xplore 15, High Wycombe, UK) approach was combined with SEM. The scanning electron microscope used a probe current of 10 nA and an accelerating voltage of 20 kV while operating in high vacuum mode (0.1 mPa).

The mineralogical composition of fabricated photocatalysts was determined by X-ray diffraction (XRD, Philips PW 1710/1820, Eindhoven, The Netherlands). Diffractograms were recorded in the range of 4–80° 2θ, counting for 1 s per 0.02° step.

Determination of the bandgap energy of photocatalysts was carried out on a Shimadzu UV-2600 (Kyoto, Japan) equipped with an integrated sphere (ISR-2600 Plus). Spectra were recorded in the range 200–800 nm.

### 2.3. Photodegradation Tests

In the thermostatic glass reactor (150 mL), a suspension of the chosen composite photocatalyst (Fe/bTiO_2_/VO_4_ or Fe/bTiO_2_/VOOH) and aqueous solution of MO was formed. During photodegradation tests, various operational parameters were varied, like photocatalyst amount (0.05–5 g/L), pollutant concentration (10–30 mg/L), irradiation time (0–120 min), lamp distance (10–30 cm), and pH (1, 3, 5, 7, 9, 11, and 13). The reaction temperature was not externally controlled and includes the experimentally observed temperature range during photocatalytic tests (approximately 20–25 °C).

The observed reactions were performed in the following way: the reaction suspension was firstly stirred in the dark for 30 min at a rate of 400 rpm on the digital magnetic stirrer ROTILABO MH 20 (Carlo Roth, Karlsruhe, Germany). After that period, a lamp with UVA and UVB (UVA:UVB = 13.6:3) radiation (Osram Vitalux 300 W, Munich, Germany) was turned on, and irradiation was initiated. Aliquots (3 mL) were sampled at the desired times, filtered through syringe filters (0.22 µm), and measured by the UV spectrometer Shimadzu 1600 (Kyoto, Japan).

Collected data from the photodegradation assay were fitted with a pseudo-first-order equation, in order to see the agreement of the experimental results with the commonly used Langmuir-Hinshelwood law [31]. Therefore, rate constants (k) and half-times (t_1/2_) of all observed reactions were calculated:

(5) $$\ln\left(\frac{C_{0}}{C}\right)=k\times t$$

where C_0_ is defined as the initial concentration of MO before achieving adsorption–desorption equilibrium in the dark, prior to irradiation, and C is the concentration of MO at chosen time t (min); *k* (min^−1^) is the reaction rate constant.

### 2.4. Computational Methods

First-principles calculations based on density functional theory were carried out using the Vienna Ab Initio Simulation Package (VASP 6.4.2) [32,33]. Exchange–correlation effects were treated within the generalized gradient approximation (*GGA*) using the Perdew–Burke–Ernzerhof (*PBE*) functional [34,35], and the projector-augmented-wave (*PAW*) method [36,37,38] was applied to represent core–valence interactions. To describe the localized 3d electrons of Fe, V, and Ti atoms, a Hubbard U correction (*DFT + U*) was applied, with U*_eff_* values of 4.0 eV for Fe, 3.2 eV for V, and 3.5 eV for Ti, ensuring a more reliable description of the electronic structure and magnetic properties. A plane-wave basis set with a kinetic energy cutoff of 520 eV was employed, ensuring total energy convergence within 1 meV/atom. For bulk structural relaxations, Brillouin-zone integration was performed using a Monkhorst–Pack k-point grid of 6 × 6 × 6, while a reduced grid, 3 × 3 × 3, was used for heterostructure calculations. Structural relaxations employed energy and force convergence thresholds of 1 × 10^−5^ eV and 0.05 eV Å^−1^, respectively. Dipole corrections were applied where necessary to account for the potential drop across the asymmetric interfaces. The planar-averaged electrostatic potential was extracted to determine the vacuum level and work function. A vacuum region of ~25 Å was added along the *z*-axis to eliminate interactions between periodic images. Visualization of the optimized structures was conducted using VESTA 3.5.8 [39].

## 3. Results and Discussion

### 3.1. Structural Properties

**Figure 3:**
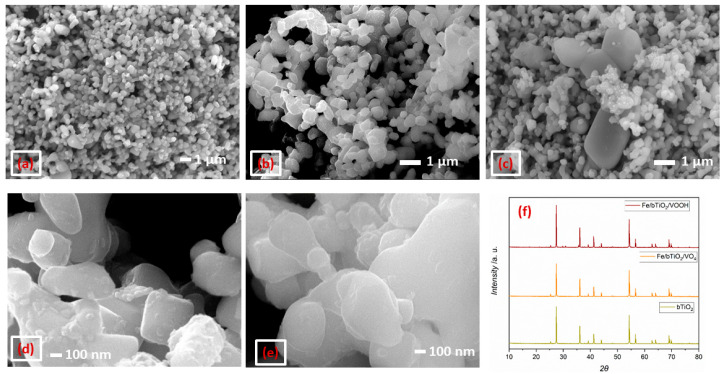
Microscans of fabricated bTiO_2_ ((**a**) 5000× magnification); Fe/bTiO_2_/VO_4_ ((**b**) 10,000× and (**d**) 50,000× magnification); and Fe/bTiO_2_/VOOH ((**c**) 10,000× magnification and (**e**) 50,000× magnification) particles; XRD scans of employed photocatalysts (**f**).

In order to obtain better insight of surface chemistry and morphology, the obtained particles underwent the SEM technique at different magnifications. The produced bTiO_2_, Fe/bTiO_2_/VO_4_, and Fe/bTiO_2_/VOOH photocatalysts’ SEM micrographs and XRD patterns are displayed in Figure 3, displaying their surface morphology and crystallographic characteristics. Figure 3a indicates that bTiO_2_ has a very porous morphology and a surface filled with microscopic fissures and fractures suitable for further chemical and thermal modification. As stated in previous work, bio-based grains are composed of rutile-phase TiO_2_, with particles diameters from 100 to 250 nm [25]. Their asymmetrical form suggests a sponge-like arrangement.

**Figure 4:**
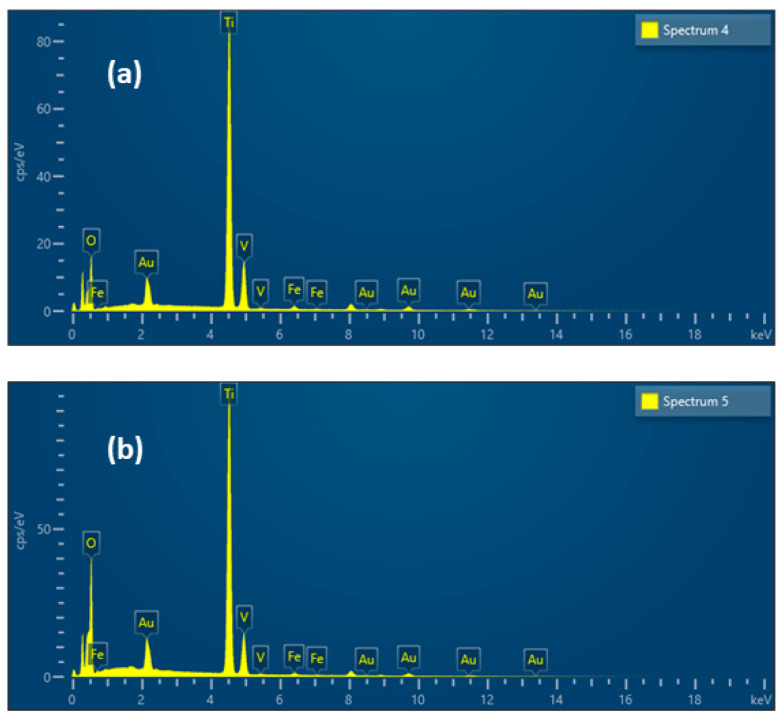
EDS spectra of Fe/bTiO_2_/VO_4_ (**a**) and Fe/bTiO_2_/VOOH (**b**).

After deposition of FeVO_4_ onto TiO_2_ grains (Figure 3b,d), several morphological changes were observed. Namely, the particles exhibit a smoother morphology with more well-defined edges compared to the starting material. Their diameter slightly decreased after calcination, ranging between 85 and 210 nm. The observed changes in morphology after modification can be associated with altered crystal growth dynamics. The presence of Fe- and V-containing phases may influence nucleation processes and limit excessive grain coalescence during thermal treatment, resulting in smoother particle surfaces and reduced particle size. In addition, organic compounds originating from the citrus extract used in the synthesis may contribute to controlled nucleation and reduced aggregation of particles. The results from EDS analysis (Figure 4a) show the following distribution of elements: 32.50 wt.% Ti, 66.65 wt.% O, 0.54 wt.% Fe, and 0.32 wt.% V.

The performed calculations revealed that 5.21 wt.% of iron vanadate was formed on the TiO_2_ surface.

Additional photocatalyst surfaces, Fe/bTiO_2_/VOOH, can be seen in Figure 3c,e. The deposited particles are evenly distributed on the base material. They mostly possess a flake-like shape and are hardly encountered as sticks. The obtained particles have a diameter range from 65 to 190 nm, making them smaller than previously synthesized photocatalysts. Four expected constituents (titanium—32.42 wt.%, oxygen—66.34 wt.%, vanadium—0.18 wt.%, and iron—1.05 wt.%) have been identified on the produced photocatalysts’ edges by EDS investigations (Figure 4b). The computations confirmed that the quantity deposited of V-substituted goethite onto TiO_2_ was 4.41 wt.%. The elemental composition obtained from EDS analysis is in qualitative agreement with the nominal amounts of Fe and V precursors used during synthesis, confirming their successful incorporation into the TiO_2_-based structure. It should be noted that EDS is a semi-quantitative technique and was used primarily to verify the presence and distribution of the elements rather than to determine exact stoichiometric ratios.

After chemical modification of the bTiO_2_ surface with additional Fe-based particles, some changes in diffractograms are observed (Figure 3f). Similar peaks for bTiO_2_ as well as for Fe/bTiO_2_/VO_4_ and Fe/bTiO_2_/VOOH were detected in our previous works, where we employed only chemical agents without a bio-based fraction for obtaining the starting material [24,25]. Those facts undoubtedly prove efficient in the synthesis of the proposed composite photocatalysts. The crystallite size was estimated using the Scherrer equation based on the XRD data: for bTiO_2_, this value is 260 nm, while Fe/bTiO_2_/VO_4_ and Fe/bTiO_2_/VOOH, the calculated crystallite size is 207 nm and 180 nm, respectively. The obtained results indicate a slight decrease in crystallite size after modification, which is consistent with SEM observations and suggests that the incorporation of additional phases affects crystal growth behavior.

**Figure 5:**
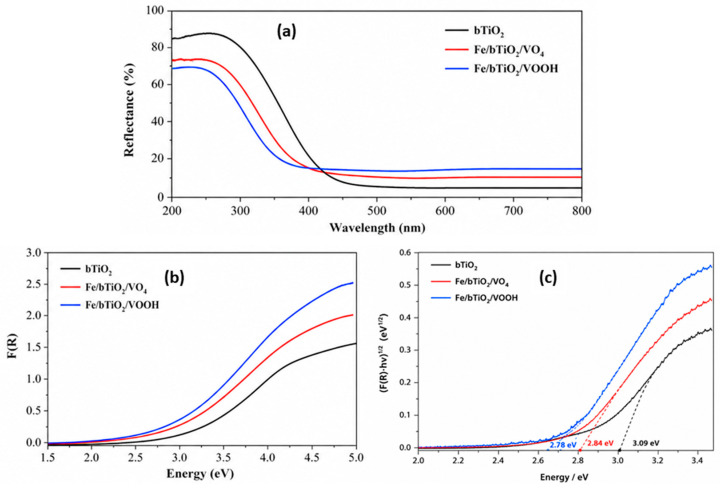
Diffuse reflectance of spectra of bTiO_2_, Fe/bTiO_2_/VO_4_, and Fe/bTiO_2_/VOOH (**a**); Kubelka–Munk-transformed spectra of bTiO_2_, Fe/bTiO_2_/VO_4_, and Fe/bTiO_2_/VOOH (**b**); Tauc plots of bTiO_2_, Fe/bTiO_2_/VO_4_, and Fe/bTiO_2_/VOOH (**c**).

The optical properties of the synthesized materials were investigated using diffuse reflectance spectroscopy (DRS), as shown in Figure 5a. Compared to pristine bTiO_2_, the modified photocatalysts exhibit enhanced absorption in the near-UV/visible region, indicating that Fe- and V-containing phases influence the optical response of the TiO_2_-based structure. The reflectance data were further transformed using the Kubelka–Munk function (Figure 5b) to obtain a quantity proportional to the absorption coefficient. Based on this transformation, Tauc plots were constructed (Figure 5c) for the estimation of bandgap energies. The improved representation of the Tauc plots, including clearly extended linear regions and extrapolation to the energy axis, allows for more reliable determination of apparent bandgap values and improves the transparency of the optical analysis.

However, it should be emphasized that these values should be interpreted as apparent optical bandgaps rather than intrinsic bandgap energies. In Fe- and V-doped TiO_2_ systems, the incorporation of transition metal dopants introduces localized electronic states within the bandgap, which can contribute to sub-bandgap absorption and lead to multiple optical transitions. Therefore, the observed absorption edge does not necessarily correspond to a single band-to-band transition. The observed red-shift and apparent bandgap narrowing are thus attributed not only to modifications in the electronic structure of TiO_2_ but also to the formation of dopant-induced intermediate energy levels and possible defect states, such as oxygen vacancies. Consequently, the optical absorption behavior represents a convolution of different electronic transitions rather than a single excitation process. This interpretation is supported by DFT results, which indicate the contribution of Fe 3d and V 3d orbitals within the bandgap region, consistent with the experimentally observed shift in optical absorption.

### 3.2. Photocatalytic Degradation

**Figure 6:**
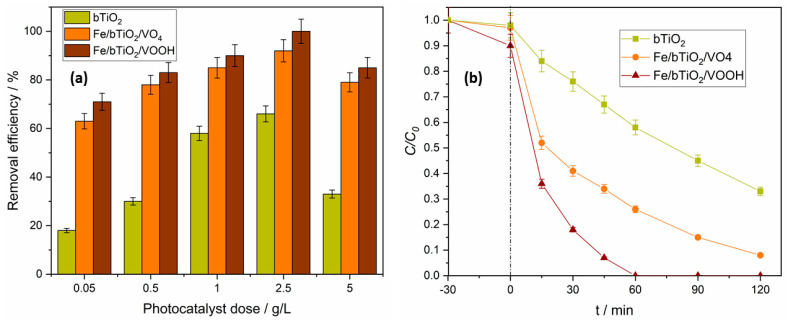
Influence of photocatalyst dose (degradation time—120 min; C_0_ (MO)—10 mg/L at room temperature) on three different photocatalysts (**a**). Comparison of efficiency toward MO degradation on fixed initial photocatalyst amount (2.5 g/L) (**b**). Initial dye concentration (degradation time—120 min, C_0_ (photocatalyst)—2.5 g/L; at room temperature). The dashed line separates the experiments performed in the dark (negative side of the x-axis) from those conducted under UV irradiation.

Figure 6a demonstrates photodegradation of MO with bTiO_2_, Fe/bTiO_2_/VO_4_, and Fe/bTiO_2_/VOOH. During reaction time, the impact of various photocatalyst dosages (0.05; 0.5; 1; 2.5; and 5 g/L) was investigated in order to determine flawless conditions.

It is revealed that Fe/bTiO_2_/VO_4_ and Fe/bTiO_2_/VOOH surpassed base bTiO_2_ (Figure 6a,b). This phenomenon could be associated to these higher functionalities on modified photocatalysts. Fe/bTiO_2_/VOOH produced results deemed roughly 8% above Fe/bTiO_2_/VO_4_ and 33% greater than bTiO_2_. It can be seen that deposition of Fe-based particles on the bTiO_2_ surfaces considerably boosted MO’s degradation efficiency.

The reduction in MO concentration was merely 18% when 0.05 g/L of bTiO_2_ was added to the mixture. The decolorization efficiency rose to 66% when the dosage of bTiO_2_ was increased from 0.05 to 2.5 g/L. Since there were fewer active sites for the photocatalytic process due to the low amount of photocatalyst, the rate of degradation was lower at lesser levels. Consequently, the decrease in catalyst loading reduced the effective surface area, thereby lowering the overall elimination efficiency.

When Fe/bTiO_2_/VO_4_ and Fe/bTiO_2_/VOOH were employed, the degradation efficiency followed same trend as for bTiO_2_, namely 63% and 83% at a 0.5 g/L dosage of photocatalysts, respectively. At 1 g/L, it was 85% and 90%, while at 2.5 g/L, it was 92% and 99%, which was the best result.

The degradation performance at 5 g/L was 33%, 79%, and 85% for bTiO_2_, Fe/bTiO_2_/VO_4_, and Fe/bTiO_2_/VOOH, respectively. Hence, 2.5 g/L of photocatalyst was suggested as the optimal dosage (Figure 6b). The chosen amount gives a sufficient number of active sites on the photocatalyst’s surface while achieving a good suspension between the aqueous pollutant solution and photocatalyst. Therefore, synergetic effects of a proper photocatalyst amount, valid irradiation penetration, and contact time of pollutants, molecules, and photocatalyst lead to improved decay of the observed contaminant [40]. The highest dosage (5 g/L) leads to a drop in system efficiency. This could possibly be explained due to coagulation and sedimentation, as well as a lower photocatalyst specific surface area and the amount of reachable active sites on the particle surface [41]. Furthermore, an excessive photocatalyst amount possibly prevented photons from penetrating, which would have decreased the activation of photocatalytic sites. Therefore, 2.5 g/L produced the optimum combination of enough active sites and effective light use, resulting in the best performance.

Lamp distance from the reactor was the next operational parameter that was investigated as well. The smallest distance (10 cm) leads to the evaporation of the polluted solution from the reactor due to an inefficient cooling system, while the largest gap between the solution and lamp causes the poorest efficiency due to the long pathway that the photon needs to travel and be included in photooxidation reactions. Therefore, the optimal distance was 20 cm, which was used in all following tests.

**Figure 7:**
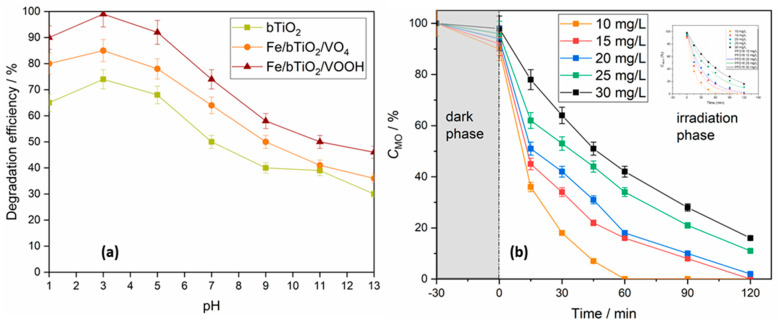
Influence of initial pH values (degradation time—120 min, C_0_ (photocatalyst)—2.5 g/L, and C_0_ (MO)—10 mg/L at room temperature) on degradation efficiency after 120 min of irradiation (**a**). Influence of starting pollutant concentration on degradation profile of MO, using 2.5 g/L Fe/TiO_2_/VOOH photocatalyst at room temperature, pH = 3.02 (**b**).

Figure 7 displays the impact of starting dye concentrations and pH levels.

Figure 7a shows how the removal of MO is affected by varying starting pH values between 1.1 and 13.2. The elimination rate showed a decreasing pattern as the initial pH levels increased. At a desirable starting pH of 3.02, 99% of the color was removed. Once the pH was raised from 3.06 up 7.11, the MO solution dropped more than 74% of its starting concentration. MO is an acid–base indicator that undergoes a structural change with the pH; above pH 4.4, it predominantly exists in its deprotonated anionic form. The negatively charged MO molecules were captured by positively charged iron nanoparticles (<pHpzc = 8) at pH values between 4.0 and 8.0. Photocatalytic composites benefited from an acidic solution to maximize the utilization of its reactive sites and prevent the accumulation of ferrous hydroxide on iron edges [42,43]. Nevertheless, raising the pH to 9.05 led to only 58% removal efficiency. This outcome is expected, as alkaline conditions shift the surface charge of the photocatalyst toward more negative values. Such a change promotes electrostatic repulsion with MO molecules, thereby hindering their adsorption onto the catalyst surface and ultimately reducing the photocatalytic efficiency. The adsorption of MO during the dark equilibrium period was found to slightly contribute (less then 8%) to the initial removal percentage, particularly at specific pH values.

Figure 7b presents the influence of various initial concentrations of MO. It is observed that with reduced starting MO concentrations, the degradation efficiency increases. Namely, raising the concentrations from 10 to 30 mg/L, after 120 min of reaction the degradation rate goes down from 99% to 84%. At an elevated level of MO (30 mg/L), a substantial fall in the rate of degradation to 84% is demonstrated. This was attributed to the fact that the amount of MO ions surpassed the photocatalysts’ considerable reactivity restriction, which therefore restrained the stimulation of the photocatalyst. The observed decrease in photocatalytic performance at higher MO concentrations can be attributed to light screening effects, which reduce photon availability at the catalyst surface. On the contrary, most MO molecules are at the photocatalyst surface and fully exposed at reduced MO concentrations (20 mg/L or 25 mg/L), resulting in an uninterrupted process with maximum performance. The photocatalyst can demonstrate its complete degrading capacity at the lowest MO concentration of 10 mg/L. Therefore, an initial MO concentration of 10 mg/L was identified as the optimal condition for achieving the highest photocatalytic degradation efficiency in this study.

The photocatalytic degradation of MO is generally reported to proceed through intermediate aromatic species prior to eventual mineralization, as described in previous studies [44,45,46].

The degree of degradation was just 2% once photolysis was carried out without the photocatalyst, with a minimal rate constant of 0.0009 min^–1^. The degradation process is likely governed by multiple oxidative pathways, including hole-driven oxidation, hydroxyl radical formation, and possible photoinduced reactions involving dissolved Fe species, as suggested in previous studies [47,48,49].

### 3.3. Kinetics of Photodegradation

Utilizing Equation (5), the gathered findings shown in Figure 7b were fitted to achieve compliance with the pseudo-first-order law. Table 1 summarizes the computed rate constants (*k*) and half-reaction durations (*t*_1/2_).

Regarding every starting amount of dye examined, significant correlation coefficients (R^2^ ≥ 0.97) were found, indicating a good fit to the pseudo-first-order (PFO) law kinetic model. The rate constant (k) nearly quadrupled (from 0.014 to 0.065 min^−1^) when the MO content decreased. This behavior can be attributed to the increased competition of dye molecules for the active sites on the photocatalyst surface and to the reduced penetration of photons caused by the higher absorbance of the solution at elevated dye concentrations [25].

### 3.4. Multistep Photodegradation

**Figure 8:**
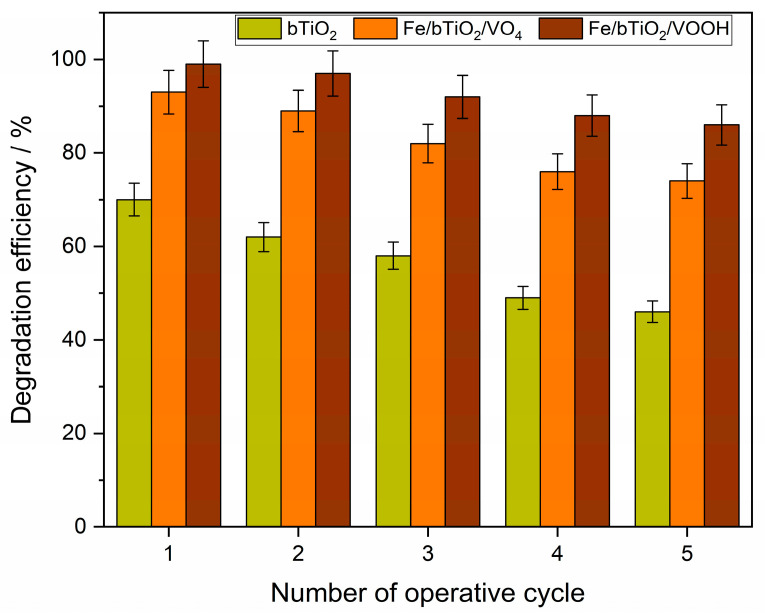
Consecutive photocatalyst cycles of MO degradation using bTiO_2_, Fe/bTiO_2_/VO_4_, and Fe/bTiO_2_/VOOH.

In an empirical investigation, photocatalysts’ versatility and durability characteristics are important in addition to their photocatalytic properties. Repetitive reuse procedures were carried out for the decomposition of MO solution over the manufactured heterojunction in order to determine the reusability of Fe/bTiO_2_/VOOH and Fe/bTiO_2_/VO_4_ composites. The degradation rate of the various cycles (up to five cycles) is displayed in Figure 8 for the operational life estimation of all three photocatalysts.

A prolonged lifespan for synthetic composites is shown by the degradation performance, which shows little variation following every degradation stage. The MO degradation efficiency dropped slightly after five cycles, indicating that the heterojunctions is highly stable in terms of photodegradation function. Importantly, iron serves as a tactical trap for photogenerated electrons and holes; by offering intermediate energy levels, it ensnares those charge carriers and considerably reduces their rate of recombination [50,51]. The carriers will have enough time to travel to the catalyst surface and initiate the redox processes required for degradation. This exceptional efficacy suggests that in terms of MO dye degradation, binding among TiO_2_ and deposited particles obtains a staggering decay rate in comparison to pure TiO_2_.

To evaluate the catalytic performance of the synthesized material, a comparison with previously reported photocatalytic systems for methyl orange (MO) degradation was performed, as summarized in Table 2. The selected studies represent various Fe-based and hybrid photocatalysts reported in the literature, enabling a comprehensive assessment of catalytic efficiency, reaction kinetics, and catalyst dosage.

It should be noted that a direct comparison between different photocatalytic systems is difficult because the reported studies were performed under different experimental conditions, including catalyst dosage, irradiation source, and initial dye concentration. Among the previously reported materials, the Fe_3_O_4_/SA/PANI/ZnO composite exhibited a relatively high apparent rate constant (k = 0.146 min^−1^), achieving 98% degradation of MO within 25 min at an initial concentration of 50 mg/L using a catalyst loading of only 0.10 g/L [52]. The enhanced catalytic activity of this material is primarily attributed to the synergistic interaction between ZnO semiconductor particles and the conductive PANI matrix, which improves electron transport and suppresses recombination of photogenerated electron–hole pairs. Bare Fe_3_O_4_ nanoparticles demonstrated a lower kinetic constant (k = 0.037 min^−1^) and required a longer reaction time of 110 min to reach 98.3% degradation efficiency, even though the experiment was conducted at a significantly higher dye concentration (1175 mg/L) [53]. This behavior highlights the limited intrinsic photocatalytic activity of Fe_3_O_4_, which mainly acts as a magnetic support rather than an efficient photocatalyst.

Carbon-based hybrid systems have also been explored to enhance photocatalytic activity [54]. The Fe_3_O_4_/GO composite achieved 99.05% MO degradation; however, the process required a long irradiation time (240 min) and exceptionally high catalyst loading (4.375 g/L). While graphene oxide improves electron mobility and surface area, the requirement for such a large amount of catalyst significantly limits the economic feasibility and scalability of this system.

A similar trend can be observed for the β-FeOOH/Fe_3_O_4_/biochar composite, which exhibited a very high apparent kinetic constant (k = 0.4087 min^−1^) and achieved 98% degradation efficiency [55]. Nevertheless, the degradation process required prolonged irradiation (5 h) and catalyst loading of 1 g/L, suggesting that adsorption processes on the porous biochar matrix significantly contribute to the overall removal efficiency rather than pure photocatalytic degradation.

Other systems reported in the literature show considerably lower photocatalytic performance. For example, the FeOOH/TiO_2_ composite achieved only 34.7% degradation after 150 min, indicating inefficient charge separation and limited catalytic activity [56]. Likewise, the ternary Fe_3_O_4_TiO_2_/MWCNTs system exhibited a very low reaction rate constant (k = 0.0085 min^−1^) and only 81% degradation efficiency after 120 min, which suggests that the addition of carbon nanotubes alone does not necessarily guarantee improved photocatalytic performance [57].

In comparison with these systems, the Fe/bTiO_2_/VOOH photocatalyst developed in the present study demonstrates highly competitive catalytic performance. The material achieved 99% degradation of methyl orange within 120 min with an apparent kinetic constant of 0.065 min^−1^ at an initial dye concentration of 10 mg/L. Although some systems exhibit higher kinetic constants, they often require significantly higher catalyst dosages or longer reaction times.

The enhanced performance of the Fe/bTiO_2_/VOOH photocatalyst may be attributed to the formation of a multi-component heterostructure, which could facilitate charge separation and interfacial electron transfer; however, this hypothesis is not directly confirmed and requires further investigation. The presence of Fe species acts as an electron mediator, while the modified TiO_2_ phase provides active photocatalytic sites. Additionally, the VOOH phase may contribute to improved light absorption and additional redox-active centers, which collectively enhance the generation of reactive oxygen species responsible for dye degradation.

Importantly, the developed catalyst provides a favorable balance between catalytic activity, degradation efficiency, and operational parameters. Compared with several literature systems that require excessive catalyst loading or extended reaction times, the Fe/bTiO_2_/VOOH system demonstrates efficient dye removal under moderate conditions, indicating strong potential for practical environmental applications.

Overall, the comparison presented in Table 2 indicates that the photocatalytic performance strongly depends on experimental conditions such as light source, pollutant type, and catalyst loading, which complicates direct comparison between studies. Nevertheless, the prepared Fe/bTiO_2_-based materials show competitive activity under the applied conditions, suggesting that the adopted synthesis approach provides an effective route for obtaining photocatalysts with promising performance.

### 3.5. Computational Results

**Figure 9:**
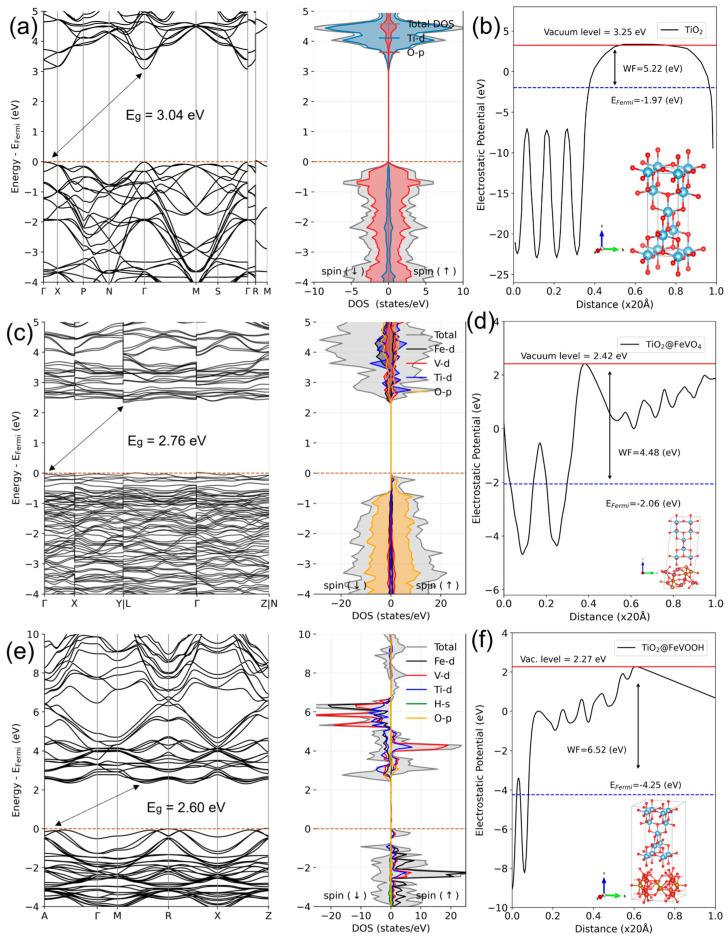
Calculated band structures and pDOS for (**a**) bTiO_2_, (**c**) FeVO_4_, and (**e**) the Fe/bTiO_2_/VO_4_ heterostructure, with the Fermi level (EF) aligned at 0 eV (shown by brown dashed lines). (**b**,**d**,**f**) Planar-averaged electrostatic potential profiles for the corresponding systems. The vacuum level (red solid line), Fermi level (blue dashed line), and calculated work functions are indicated. Insets show the optimized crystal structures of the respective systems. In the inset structures, red atoms represent oxygen (O), blue atoms represent titanium (Ti), white atoms represent hydrogen (H), and golden-colored atoms represent iron (Fe).

The electronic structure and interfacial charge-transfer properties of TiO_2_, TiO_2_/FeVO_4_, and TiO_2_/FeVOOH were investigated using density functional theory (DFT) to elucidate their roles in photocatalytic activity toward methyl orange degradation. The calculated band structures, projected density of states (pDOS), and planar-averaged electrostatic potentials are shown in Figure 9. Pristine TiO_2_ (Figure 9a) exhibits a wide bandgap of 3.04 eV, which limits its visible-light absorption. The valence band maximum (VBM) is predominantly derived from O-2p orbitals, whereas the conduction band minimum (CBM) is mainly composed of Ti-3d states, indicating a typical O-Ti charge-transfer character. The planar-averaged electrostatic potential (Figure 9b) reveals a vacuum level of 3.25 eV and a Fermi level (E*_Fermi_*) of −1.97 eV, corresponding to a work function of 5.22 eV, which reflects moderate electron affinity but does not favor efficient charge separation. In contrast, TiO_2_@FeVO_4_ (Figure 9c) shows a reduced bandgap of 2.76 eV, indicating enhanced visible-light absorption. The pDOS indicates that Fe-3d and V-3d orbitals contribute significantly near both the VBM and CBM, introducing additional electronic states that facilitate charge excitation and transfer. This modification improves the material’s ability to generate photocarriers under visible light, which is critical for dye degradation processes. The electrostatic potential profile (Figure 9d) shows a vacuum level of 2.42 eV and a E*_Fermi_* of −2.06 eV, yielding a work function of 4.48 eV, suggesting an increased tendency for electron donation compared to pristine TiO_2_. For the TiO_2_/FeVOOH heterostructure (Figure 9e), the electronic structure undergoes further modification, with a bandgap of 2.60 eV. The pDOS analysis reveals pronounced hybridization among Ti-3d, Fe-3d, and V-3d orbitals, resulting in increased electronic delocalization and the formation of interfacial states near the Fermi level. This orbital interaction facilitates improved charge transport across the interface. As shown in Figure 9f, the electrostatic potential exhibits a vacuum level of 2.27 eV and a deeper Fermi level at −4.25 eV, resulting in an increased work function of 6.52 eV. The variation in work functions among the three systems establishes a clear driving force for interfacial charge transfer. Specifically, the lower work function of TiO_2_ relative to TiO_2_/FeVOOH promotes electron migration from TiO_2_ to the Fe-containing component until Fermi-level alignment is achieved. This redistribution of charge generates an internal electric field at the heterointerface, as evidenced by the potential gradient in the electrostatic profiles. Consequently, photogenerated electrons preferentially accumulate on the Fe-containing phase, while holes remain in the TiO_2_ region, effectively enhancing charge separation and suppressing electron–hole recombination. Overall, the formation of the TiO_2_/FeVO_4_ and TiO_2_/FeVOOH heterostructures not only narrows the bandgap and enhances visible-light absorption but also establishes a built-in electric field that facilitates directional charge transfer. These combined effects are expected to significantly improve the generation of reactive species (•OH and ${•O}_{2}^{-}$), thereby enhancing the photocatalytic degradation efficiency of methyl orange. Moreover, this behavior confirms the formation of a type-II heterojunction, which is highly advantageous for photocatalytic applications.

These results are important not only from the standpoint of band structure engineering but also in terms of interfacial energetics governing photocatalysis in water. The Fe- and V-based heterostructures modify the electronic structure in a way that directly influences adsorption of MO, interfacial charge transfer, radical generation, and degradation kinetics. Thus, the improved photocatalytic behavior can be understood as a consequence of both favorable electronic alignment and a tunable interfacial free-energy landscape shaped by surface composition, solvent interactions, and entropy-related effects. By combining photocatalytic experiments with DFT analysis, this study therefore provides deeper insight into how interfacial energetics and entropy-related factors can be tuned to improve the catalytic efficiency of TiO_2_-based systems for aqueous environmental remediation.

## 4. Conclusions

The aim of this study was to effectively degrade MO dye from a water solution by employing fabricated photocatalysts under UV/Vis light irradiation. Utilizing bio-based bTiO_2_ as core nanoparticles for the development of composites, a green synthesis method was effectively used to create a novel Fe/bTiO_2_/VOOH and Fe/bTiO_2_/VO_4_ photocatalysts. In the context of environmental protection, Fe/bTiO_2_/VOOH and Fe/bTiO_2_/VO_4_ show exceptional efficiency in the photocatalytic degradation of MO through the generation of *OH radicals. The incorporation of Fe and V alters the valence and conduction bands, resulting in a reduced bandgap and a modified work function. This suggests that enhanced visible light absorption and improved charge carrier separation were key factors for increasing photocatalytic efficiency.

Using Fe/bTiO_2_/VOOH at 2.5 g/L, a decolorization rate of 99% was attained during the 120 min period, degrading 82% of the starting MO quantity, initiated by sunlight-imitating radiation. Compared to Fe/bTiO_2_/VO_4_’s efficiency of 92%, base bTiO_2_ showed only 33%, at the same reaction conditions. The photocatalysis aligned to pseudo-first-order kinetics, and the composite’s higher photocatalytic effectiveness was supported by the calculated rate constants (k) values.

Throughout five successive reuses, reusability studies showed that the composite materials have a high degree of resilience, maintaining over 80% of its original efficacy. The composite’s capacity for practical use is confirmed by the sustained functionality, which shows little decay in the structure.

Fabricated hybrid materials could pose as a solution for the treatment of fast-growing, highly polluted industrial wastewaters enriched with organic matter, mostly dyes.

## Figures and Tables

**Table 1:** Kinetics parameters of MO photodegradation with Fe/TiO_2_/VOOH.

**C_0_(MO) (mg/L)**	*k* ± SD * (min^−1^)	*t*_1/2_ (min)	R^2^
10	0.065 ± 0.0061	10.82	0.99
15	0.053 ± 0.0039	13.19	0.98
20	0.043 ± 0.0031	16.04	0.97
25	0.020 ± 0.0014	34.53	0.98
30	0.014 ± 0.00029	46.38	0.99

* SD—standard deviation.

**Table 2:** Brief comparison of MO’s photocatalytic decay variables.

C_0_ (mg/L)	Photocatalyst	Light Source	*k* (min^−1^)	Time (min)	Efficiency (%)	Amount (g/L)	Ref.
50.0	Fe_3_O_4_/SA/PANI/ZnO	xenon lamp (250 W)	0.146	25	98	0.10	[52]
1175	Fe_3_O_4_	low-pressure UV lamp (4 W)	0.037	110	98.3	0.020	[53]
10	Fe_3_O_4_/GO	two UV lamps (30 W)	-	240	99.05	4.375	[54]
100	β-FeOOH/Fe_3_O_4_/biochar	xenon lamp (350 W)	0.4087	300	98	1	[55]
10	FeOOH/TiO_2_	LED light (5 W)	-	150	34.7	-	[56]
10	Fe_3_O_4_/TiO_2_/MWCNTs	UV–Vis lamp (500 W)	0.0085	120	81	0.20	[57]
10	Fe/bTiO_2_/VOOH	Sun-imitating lamp (300 W)	0.065	120	99	2.5	Our study
	Fe/bTiO_2_/VO_4_		0.058		92		

## Data Availability

The data presented in this study are available on request from the corresponding author.
